# Prolactin, Estradiol and Testosterone Differentially Impact Human Hippocampal Neurogenesis in an *In Vitro* Model

**DOI:** 10.1016/j.neuroscience.2019.12.021

**Published:** 2021-02-01

**Authors:** Demelza M. Smeeth, Ioanna Kourouzidou, Rodrigo R.R. Duarte, Timothy R. Powell, Sandrine Thuret

**Affiliations:** a Department of Basic and Clinical Neuroscience, Institute of Psychiatry, Psychology and Neuroscience, King’s College London, 125 Coldharbour Lane, London SE5 9NU, UK; b Social, Genetic and Developmental Psychiatry Centre, Institute of Psychiatry, Psychology and Neuroscience, King’s College London, London SE5 8AF, UK; c Department of Neurology, University Hospital Carl Gustav Carus, Technische Universität Dresden, Germany

**Keywords:** 4-OHT, 4-hydroxytamoxifen, AHN, adult hippocampal neurogenesis, AR, androgen receptor, BrdU, Bromodeoxyuridine, DCX, Doublecortin, ESR1, Estrogen Receptor 1, ESR2, Estrogen Receptor 2, HPC, hippocampal progenitor cell, ICC, immunocytochemistry, MAP2, microtubule-associated protein 2, PRLR, prolactin receptor, hippocampal neurogenesis, prolactin, estradiol, testosterone, neural stem cells

## Abstract

Previous studies have indicated that sex hormones such as prolactin, estradiol and testosterone may play a role in the modulation of adult hippocampal neurogenesis (AHN) in rodents and non-human primates, but so far there has been no investigation of their impact on human hippocampal neurogenesis. Here, we quantify the expression levels of the relevant receptors in human post-mortem hippocampal tissue and a human hippocampal progenitor cell (HPC) line. Secondly, we investigate how these hormones modulate hippocampal neurogenesis using a human *in vitro* cellular model. Human female HPCs were cultured with biologically relevant concentrations of either prolactin, estradiol or testosterone. Bromodeoxyuridine (BrdU) incorporation, immunocytochemistry (ICC) and high-throughput analyses were used to quantify markers determining cell fate after HPCs were either maintained in a proliferative state or allowed to differentiate in the presence of these hormones. In proliferating cells, estrogen and testosterone increased cell density but had no clear effect on markers of proliferation or cell death to account for this. In differentiating cells, a 3-day treatment of prolactin elicited a transient effect, whereby it increased the proportion of microtubule-associated protein 2 (MAP2)-positive and Doublecortin (DCX)-positive cells, but this effect was not apparent after 7-days. At this timepoint we instead observe a decrease in proliferation. Overall, our study demonstrates relatively minor, and possibly short-term effects of sex hormones on hippocampal neurogenesis in human cells. Further work will be needed to understand if our results differ to previous animal research due to species-specific differences, or whether it relates to limitations of our *in vitro* model.

## Highlights

• Human hippocampal progenitor cells (HPCs) and tissue express classical sex hormone receptors.
• Prolactin does not impact human HPCs maintained in a proliferative state.
• Prolactin increases neuronal differentiation of human HPCs only in the short term.
• Estradiol and testosterone both increase the cell density of proliferating HPCs.
• Estradiol and testosterone have no observed effect on differentiating HPCs.

## Introduction

The adult brain of many vertebrate species contains populations of multipotent stem cells that can give rise to new neuronal, astrocytic and oligodendrocytic lineages throughout the lifespan (Gage, 2002, Alunni and Bally-Cuif, 2016, Paredes et al., 2016). Early rodent studies identified the hippocampus as one region which possesses a resident pool of stem and progenitor cells (Altman and Das, 1965). These reside in the subgranular zone of the dentate gyrus, in a thin layer below the granule cell layer (Knoth et al., 2010). The resident stem and progenitor cells self-renew and differentiate into neurons throughout the lifespan in a constitutive manner under normal physiological circumstances (Kempermann et al., 2015). While this process can also produce a substantial number of astrocytes and oligodendrocytes (Morrens et al., 2012), research has predominately focused on the production of neurons, known as adult hippocampal neurogenesis (AHN). In the neurogenic process, newly differentiated neurons migrate into the granule cell layer and extend their axons into the CA3 region of the hippocampus (Knoth et al., 2010). Due to the inherent difficulties in studying AHN in humans, much of what we know about the process has been elucidated from mammalian animal studies, although there appear to be similarities between species (Knoth et al., 2010, Aimone et al., 2014).

There is clear evidence that AHN exists in rodents and non-human primates, and the existence of AHN in humans has been recently controversial, however many studies have found strong evidence for its existence (Eriksson et al., 1998, Spalding et al., 2013, Boldrini et al., 2018, Kempermann et al., 2018, Moreno-Jiménez et al., 2019). Due to limited studies focusing on human AHN and the common methods of studying AHN, we know very little about the modulatory mechanisms of human AHN which leaves a large gap in our collective knowledge.

The precise functions of AHN are still under investigation, although many studies support the involvement of AHN in the hippocampus’ role in learning, memory, and mood regulation (Christian et al., 2014, Gradari et al., 2016, Anacker and Hen, 2017, Toda and Gage, 2018). Indeed, ablation of AHN through genetic or irradiation techniques can produce deficits in learning and memory in animal studies involving fear condition and long-term memory retention, although this finding is not consistent across all studies (Winocur et al., 2006, Ben Abdallah et al., 2013, Park et al., 2015). It also produces deficits in discriminating similar objects, contexts or locations suggesting impaired pattern separation (Clelland et al., 2009, Kheirbek et al., 2012). In addition to this continuous memory-modulating process, AHN may also rescue neuronal loss following injury. Indeed AHN appears to be upregulated in response to injuries such as induced excitotoxicity to model epilepsy, stroke, traumatic brain injury and chronic alcohol consumption (Parent, 2002, Sun, 2014, Nickell et al., 2017, Wu et al., 2017). Finally, AHN appears to be involved in mood control and has been postulated to be impaired in human psychiatric disorders such as major depressive disorder and bipolar disorder (Kang et al., 2016, Boldrini et al., 2019) as well as a variety of animal models of depression (Spritzer et al., 2011, Chetty et al., 2014, Anacker et al., 2018, Dioli et al., 2019). Therefore, a more thorough knowledge of the control of AHN is key to understanding many hippocampal functions and cognitive processes.

Due to vascularization of the hippocampal neurogenic niche and the impact of the systemic environment on neural stem cells and AHN (Licht and Keshet, 2015, Smith et al., 2018), changes in circulating sex hormones have the potential to modulate AHN and hippocampal volume (Mahmoud et al., 2016, Smeeth et al., 2017). Indeed, circulating prolactin, estradiol and testosterone have all been shown to be able to cross the blood–brain-barrier (Pardridge and Mietus, 1979, McCall et al., 1981, Walsh et al., 1987, Brown et al., 2015) and their respective receptors have been detected in or around the dentate gyrus, at least in rodent tissue (Shughrue et al., 1997, Weiland et al., 1997, Österlund et al., 2000, Shingo et al., 2003, Milner et al., 2005, Milner et al., 2005, Perlman et al., 2005, Herrick et al., 2006, González et al., 2007, Nogami et al., 2007, Mak and Weiss, 2010). This indicates that sex hormones have the potential to access and act upon the neurogenic niche. It is unclear what the function of hormonal control of AHN may be, but it would account for the changes in AHN which occur in times of great hormonal change such as pregnancy, peripartum and the menopause (Shingo et al., 2003, Pawluski and Galea, 2007, Schmidt et al., 2015).

Prolactin is a peptide sex hormone which is elevated in pregnancy, lactation and following stressors (Sassin et al., 1972, Bonnar et al., 1975, Lennartsson and Jonsdottir, 2011). Some rodent studies indicate a proliferative and neurogenic effect of prolactin on both resident and isolated dentate gyrus neural stem cells (Mak and Weiss, 2010, Walker et al., 2012), whilst others have shown no effect of the hormone (Shingo et al., 2003, Mak et al., 2007, Wagner et al., 2009). Furthermore, prolactin can diminish chronic stress-induced deficits in hippocampal neurogenesis (Torner et al., 2009). Studies concerning the control of subventricular zone (SVZ) neurogenesis by prolactin have been more consistent, with multiple reports that prolactin increases the proliferation and neuronal differentiation of SVZ neural stem cells both *in vivo* or *in vitro* (Shingo et al., 2003, Mak and Weiss, 2010, Wang et al., 2013), while disrupted prolactin release inhibits SVZ neurogenesis *in vivo* (Larsen and Grattan, 2010). The sole study concerning the impact of prolactin upon human neural stem cells was performed using radial glia-like cortical progenitor cells isolated from a fetal brain and observed a proliferative effect of prolactin alongside a dose-dependent effect of prolactin upon differentiation into astrocytic or neuronal lineages (Pathipati et al., 2011).

Steroid hormones like estradiol and testosterone have also been investigated as modulatory factors of AHN. Ovariectomy leading to estradiol deficiency produces a short-term reduction in rat hippocampal proliferation and increased pyknosis (Green and Galea, 2008, Lagace et al., 2007, Tanapat et al., 1999, Tanapat et al., 2005). Estradiol supplementation in ovariectomized rats rescues the loss of hippocampal cell proliferation in the short term, but this is highly dependent on the dose, age, sex, parity and timepoint studied (Barha and Galea, 2011, Barha et al., 2009, Chiba et al., 2007, Galea et al., 2013, Green and Galea, 2008, Spritzer and Galea, 2007, Tanapat et al., 1999, Tanapat et al., 2005). The impact of estradiol on the later neurogenic stages has been less well studied but it appears that estradiol often has no effect and any increase in newborn neurons is suggested to be the result of an earlier push in cell proliferation (Tanapat et al., 2005, Green and Galea, 2008). Rodent *in vitro* studies also support a role for estradiol in hippocampal neurogenesis modulation. The administration of estradiol to embryonic and juvenile rat hippocampal NSCs increases both cell proliferation and neuronal differentiation (Brännvall et al., 2002, Chiba et al., 2007, Zhang et al., 2016) but decreases the proliferation of adult rat NSCs with no impact on differentiation (Brännvall et al., 2002). Finally, there is evidence that estradiol can impact human NSCs, although not of a hippocampal origin, as it increases proliferation of human embryonic cortically-derived NSCs and human iPSC-derived cortical NSCs (Wang et al., 2008, Shum et al., 2015).

Testosterone may also impact AHN, although its effect has been less extensively studied than estradiol. Once within its target tissue, testosterone can induce a response by binding directly to the androgen receptor (AR) or through metabolization to further sex steroid species including estradiol (Luu-The and Labrie, 2010). Multiple rodent studies have shown that reduction in circulating testosterone though castration decreases cell survival in the postnatal rat and mouse dentate gyrus, without impacting cell proliferation (Spritzer and Galea, 2007, Benice and Raber, 2010, Spritzer et al., 2011, Wainwright et al., 2011, Hamson et al., 2013) and this effect is moderated by the AR (Hamson et al., 2013). However, this finding may be limited to studies with higher doses of testosterone and young animals (Spritzer and Galea, 2007, Buwalda et al., 2010, Spritzer et al., 2011, Carrier and Kabbaj, 2012, Duarte-Guterman et al., 2019). Finally there is evidence that testosterone can impact neural stem cells directly, leading to increased proliferation and neuronal differentiation of both mouse NSCs and human iPSC-derived NSCs (Quartier et al., 2018, Ransome and Boon, 2015a, Ransome and Boon, 2015).

The present study attempts to extend our understanding on the effects of the sex hormones prolactin, estrogen and testosterone on hippocampal neurogenesis in three key ways. First, we confirm and compare the expression of the sex hormone receptors, Estrogen Receptor 1 (*ESR1*), Estrogen Receptor (*ESR2*), *AR* and prolactin receptor (*PRLR*) both in the human post-mortem hippocampal tissue and in human HPCs. Second, we examine the effects of sex hormones on the neurogenic process in a human hippocampal cellular system, both while hippocampal stem cells are proliferating, and whilst they are differentiating. Third, we test a wide range of hormone concentrations which encompass those found throughout the human lifespan, including during pregnancy. Overall, our study demonstrates that while steroid hormones have similar impacts on proliferative HPCs, leading to increased cell density, prolactin has only a time-dependent effect on neuronal differentiation in human HPCs.

## Experimental procedures

### *In vitro* model of hippocampal neurogenesis

An existing human fetal hippocampal multipotent progenitor cell line, HPCOA07/03 (ReNeuron, UK), was used to model human hippocampal neurogenesis *in vitro*. This cell line has been described previously (Johansson et al., 2008, Anacker et al., 2011, Powell et al., 2017). These hippocampal progenitor cells (HPCs) were sourced from a terminated 12-week old female fetus in accordance with UK and USA ethical and legal guidelines. The cells were infected with a retroviral vector pLNCX2, encoding the c-MycER^TAM^ transgene construct to generate a conditionally immortalized cell line. This transgene is solely activated by the synthetic steroid 4-hydroxytamoxifen (4-OHT) and the cells can be cultured continuously without differentiation in the presence of 4-OHT, basic fibroblast growth factor (bFGF) and epidermal growth factor (EGF). Removal of growth factors and 4-OHT from the culture medium allows the cells to differentiate into neurons, oligodendrocytes and astrocytes. Following 7 days of differentiation, the resulting cell population is composed of 35% doublecortin (DCX)-positive immature neuroblasts, 25% microtubule-associated protein 2 (MAP2)-positive neurons, 27% S100β-positive astrocytes and 2% O1-positive oligodendrocytes (Anacker et al., 2011). The remaining population maintain a neural progenitor cell phenotype. Further immunocytochemical analysis has shown that the MAP2-positive population co-stain for PROX1, a marker of dentate gyrus neurons (Powell et al., 2017). We expect that the proportions of stained cells will differ slightly in these assays due to small differences in cell culture conditions and the use of automated cellular quantification here.

HPCs were routinely cultured in Nunclon flasks (Thermo Scientific, 156367/156499), coated with 23 µg/ml laminin from Engelbreth–Holm–Swarm murine sarcoma basement membrane (Sigma, L2020) in PBS (Gibco, 18912-014) for 1–24 h to aid cell adhesion. HPCs were maintained in chemically-defined proliferation medium and incubated at 37 °C, 5% CO_2_ and sufficient humidity. Proliferation medium was composed of Dulbecco’s modified Eagle’s Medium Nutrient Mixture F-12 Ham (DMEM-F12; Sigma–Aldrich, D6421 or D6434) supplemented with 0.03% human albumin solution (Zenalb, 20), 100 μg/ml human apo-transferrin (Sigma, T1147), 16.2 μg/mL human putrescine DiHCl (Sigma, P5780), 5 μg/mL human recombinant insulin (Sigma, I9278), 60 ng/mL progesterone (Sigma, P8783), 2 mM l-glutamine (Sigma, G7513), 40 ng/mL sodium selenite (Sigma, S9133), 10 ng/mL human bFGF (Peprotech, AF 100-15-500), 20 ng/mL human EGF (Peprotech, EC 100-18B) and 100 nM 4-OHT. DMEM-F12 containing Phenol Red (D6421) was used when culturing HPCs for gene expression analysis and prolactin assays, while DMEM without Phenol Red (D6434) was used when culturing HPCs for estradiol and testosterone assays due to the estrogenic activity of Phenol Red. HPCs were passaged when they became 80–90% confluent and cultures were regularly checked for mycoplasma infection.

### Assessing the expression of sex hormone receptors in the hippocampus

*HPCs:* We previously compared expression profiles of the human hippocampal progenitor cell line (HPCOA07/03) when cells were proliferating as part of a 3-day protocol and after a subsequent 7-day differentiation protocol (Powell et al., 2017). Gene expression was assayed using the Illumina Human HT-12 v4 Expression BeadChip (Illumina Inc., California, U.S.), and processed using the Lumi (Bioconductor) package in R, as described previously (Powell et al., 2017). Here, we compare expression differences between sex hormone receptors when 12 subcultures are grown in control conditions, either whilst proliferating or differentiating.

*Post-mortem brain*: To determine expression levels of sex hormone receptors in the hippocampus, we analyzed RNA-sequencing data openly accessible from the Stanley Neuropathology Consortium (Kim and Webster, 2010). This consists of a collection of 60 matched brain samples from hippocampus and FC, 15 each from subjects diagnosed with bipolar disorder, schizophrenia, or major depression, and unaffected controls. For the purposes of this work, we only utilized data from the control cohort. This sample consisted of 6 females and 11 males, who collectively had an average age of 48.06 years (S.D. = 10.66).

RNA-seq reads were trimmed using Trimmomatic 0.36 (Bolger et al., 2014), where sequences were pruned of Illumina adaptors, low quality bases (leading/trailing sequences with phred score <3, or those with average score <15 every four bases), or reads below 36 or over 53 bases in length. Kallisto was used to align these reads to the Ensembl genome (build 38, v93) (Bray et al., 2016). Kallisto output files were imported into R and raw counts were normalized using DESeq2 (Love et al., 2014).

### Hormone treatments

Lyophilized human recombinant prolactin (Peprotech, 100-07) was reconstituted in sterile PBS to produce stock concentrations of 100 μg/ml. Human albumin solution (Zenalb, 20) was added as a carrier protein at a final concentration of 0.1% for stability during long-term storage. Estradiol (Sigma–Aldrich, E2257) was dissolved in sterile 100% ethanol (Sigma–Aldrich, 51976) and further diluted in DMEM-F12 to reach a stock concentration of 20 μg/ml. Testosterone (Sigma–Aldrich, T1500) was dissolved in a sufficient quantity of sterile 100% ethanol to obtain a final concentration of 0.5 mg/ml. Stock hormone solutions were aliquoted in working volumes, stored at −20 °C and never refrozen once thawed.

The range of hormone concentrations used was chosen to encompass those found across the human lifespan including pregnancy when plasma hormones are greatly elevated. One supra-physiological concentration was also chosen. Prolactin treatments: 0, 0.1, 1, 10, 100, 500 or 1000 ng/ml. All were created with an identical final concentration of 0.001% human albumin solution. Estradiol treatments: 0, 0.01, 0.1, 1, 5, 25 or 75 ng/ml. All treatments were created with an identical final concentration of 0.0075% ethanol. Testosterone treatments: 0, 0.01, 0.1, 1, 5, or 10 ng/ml. All treatments were created with an identical final concentration of 0.002% ethanol.

### Cellular assays

**Fig. 1:**
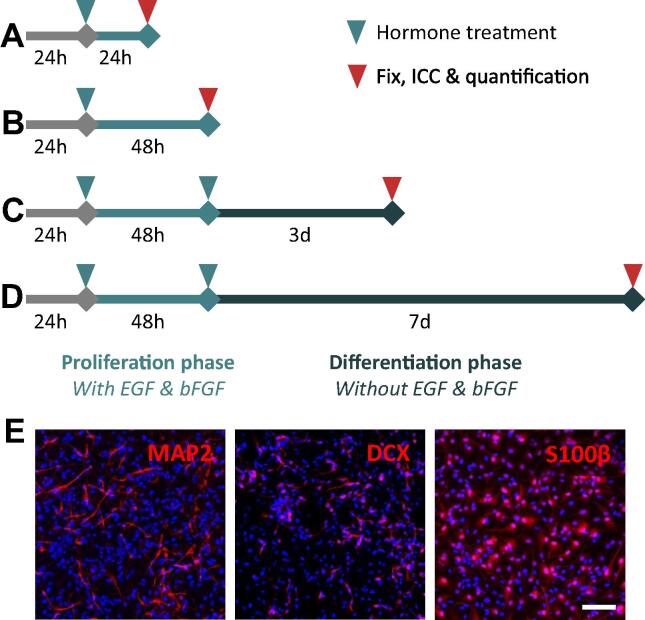
Schematics of the cellular assays performed to examine the effect of prolactin upon proliferating and differentiating hippocampal progenitor cells (HPCs). Schematics indicate the timing of treatments, and length of proliferation and differentiation phases in days. (**A**, **B**) Cells are cultured in media containing 4-OHT and growth factors (EGF and bFGF) for the duration of the proliferation phases (first 72 h). After 24 h cells are treated with either prolactin, estradiol or testosterone. After 24 h or 48 h cells are fixed and processed for ICC and quantification of cellular markers. (**C, D**) After the initial proliferation phase, cells undergo two full media washes with differentiation media (lacking EGF, bFGF and 4-OHT) and are cultured for the remaining assay tie in differentiation media containing treatment. (**E**) Three days after the initiation of differentiation, cells are supplemented with an additional hormone treatment to ensure prolactin levels remain at the target concentration throughout the differentiation phase. (**E**) Representative images of HPCs allowed to differentiate for 7 days, illustrating the ability to produce DCX and MAP2-positive neurons and S100β-positive astrocytes. DAPI nuclear staining shown in blue. Scale bar = 100 μm.

To disentangle the effects of a hormone treatment on proliferative HPCs versus differentiating HPCs, two assay types were used. Assay timelines are depicted in Fig. 1. Proliferative assays maintained cells in a proliferative state to examine treatment effects solely on proliferative aspects of cell fate. Differentiation assays were used to examine the treatment effects on HPCs allowed to differentiate. Hormones treatments were added to both the initial proliferative phases as well as the later differentiating phase to mimic the presence of hormones throughout the lifespan of the HPC in the neurogenic niche. For all assays, cells were seeded on laminin coated Nunclon 96-well plates (Thermo, 167008) at a density of 1.2 × 10^4^ cells/well in 100 µl proliferation medium as described above, except 4-OHT was omitted for estradiol and testosterone assays due to its estrogenic activity. All conditions were performed in technical triplicate on each plate and each “biological replicate” represents a subculture of cells obtained from a different passage. For all experiments, cells were used between passages 14 and 22 for consistency.

*Proliferation assays*: Twenty four hours after seeding, the proliferation medium was replaced entirely. This new medium contained either the relevant hormone or vehicle treatment as well as with EGF and bFGF (and 4-OHT for prolactin assays). HPCs were incubated with the hormone for 24 or 48 h. Cells were treated with 10 µM bromodeoxyuridine (BrdU; Sigma, B9285) for the last 4 h of the assay before being washed with warm DMEM-F12, and fixed through the addition of 50 µl/well freshly-thawed room temperature 4% paraformaldehyde (PFA, Alfa Aesar 43368) in PBS (Sigma, P4417) for 20 min at room temperature in the dark. Fixed cells were subsequently washed three times with PBS and stored at 4 °C in 0.05% sodium azide (Sigma, S8032) in PBS wrapped in parafilm (Sigma, P7793) to prevent drying out.

*Differentiation assays:* After seeding, HPCs were allowed to incubate for 24 h before replacement of proliferation medium with fresh proliferation medium containing the relevant treatment as above. Following 48 h of incubation, HPCs were washed twice with differentiation medium (proliferation medium without 4-OHT, EGF or bFGF) to remove any remaining 4-OHT or growth factors. Cells were subsequently treated with the appropriate treatment diluted in differentiation medium. Cells were incubated further for either 3 or 7 days. If BrdU incorporation was performed, HPCs were treated with 10 µM BrdU for the last 4 h of incubation. Cells were fixed with PFA and stored as described previously.

### Immunocytochemistry

ICC was carried out to analyze the fate and morphology of control and hormone-treated HPCs. Following fixation, BrdU treated wells were incubated with 2 N hydrochloric acid in order to denature DNA strands and neutralized with 0.1 M sodium borate buffer. Cells were subsequently washed with PBS. All cells were blocked with blocking solution composed of 5% normal donkey serum (Sigma, D9663) and 0.3% Triton X-100 (Sigma, T9284) in PBS for 1 h.

Primary antibodies were diluted to the appropriate concentration in blocking solution and added to the cells overnight at 4 °C. All plates contained HPCs grown in control conditions which were exempt from primary antibody staining to be used as a benchmark for background fluorescence for later analysis. Antibodies were chosen to identify cells in the various stages of proliferation, apoptosis and differentiation. Primary antibodies used to label cells after proliferation assays: rat anti-BrdU (1 in 500; Serotec, OBT0030CX), rabbit anti-KI67 (1 in 500; Abcam, Ab15580), mouse anti-KI67 (1 in 800; Cell Signaling, 9449) and rabbit anti-CC3 (1 in 500; Cell Signaling, 9664). Primary antibodies used to label cells after differentiation assays: rat anti-BrdU, rabbit anti-KI67, mouse anti-KI67, rabbit anti-CC3, mouse anti-Nestin (1in 1000; Chemicon, mab5326), rabbit anti-DCX (1 in 500, Abcam, Ab18723), mouse anti-MAP2 (1 in 500; Abcam, Ab11267) and rabbit anti-S100β (1 in 500; Dako, Z0311). Cells were washed thoroughly with PBS and further incubated with blocking solution for 30 min. Secondary antibodies were diluted in blocking solution and added to cells for 2 h. Secondary antibodies: Alexa 488 donkey anti-rat (Life Technologies, A-21208), Alexa 488 donkey anti-rabbit (Life Technologies, A-21206), Alexa 488 donkey anti-mouse (Life Technologies, A-21202), Alexa 555 donkey anti-rabbit (Life Technologies, A-231572) and Alexa 555 donkey anti-mouse (Life Technologies, A-31570) all at 1 in 500. Cells were washed with PBS, nuclei were stained with 300 nM DAPI for 5 min and washed again with PBS. Cells were stored in 0.05% sodium azide in PBS at 4 °C wrapped in foil.

### Automated quantification of immunofluorescent staining

Automatic and unbiased quantification of cellular antibody staining was performed using the semi-automated high-throughput Thermo Scientific CellInsight CX5 High Content Screening Platform (Thermo Scientific) alongside the HCS Studio Cell Analysis Software (Thermo Scientific). Protocols were developed using the software’s Cell Health Profiling BioApplication, which identifies individual cells based on nuclear staining and quantifies the intensity of fluorescent staining in user-defined regions with respect to this. All parameters required to do this were defined manually and kept constant across entire experiments, but differed between different cellular assays. On rare occasions, parameters varied between biological replicates to account for differences in overall immunofluorescent staining brightness.

Briefly, images were taken with a 10× objective using the DAPI stain to autofocus. Exposure times for each channel were manually defined to ensure a good signal to background ratio using the unstained control wells as a guide for background fluorescence. A selection of at least three stained and three unstained wells were imaged and used as representative images on which to optimize the remainder of the protocol. Images underwent a background removal step for all channels using the software-recommended parameters for intensity-relevant measures. Individual cells were automatically identified using the DAPI-positive nuclei. Smoothing, threshold and segmentation parameters were adjusted to ensure individual nuclei were outlined accurately. Nuclei were filtered by size to exclude DAPI-positive debris and unsegmented nuclei. Nuclei were also excluded if they touched the image field border.

Parameters were defined to locate and quantify the cellular markers of interest depending on their location in relation to the nucleus. Nuclear proteins were located with a round target overlapping the nuclear outline, while cytoplasmic stains were located with a ring target, whose inner boundary overlaps slightly with the nucleus. Positive or negative staining for each marker in individual cells was defined based upon the average intensity of fluorescent signal across the set target region and was determined by a manually defined threshold. Overall, this method produces the proportion of DAPI-positive cells which stain positively for each cellular marker of interest. The same parameters were used on all conditions within each experiment and all biological replicates. Up to 15 fields of view were imaged per well, located in the center of the well to ensure good cell coverage. This quantified 4000–10,000 cells per well depending on the cellular assay used.

### Automated neurite outgrowth analysis

This was performed to quantify the neurite outgrowth and branching of neuronal cells after 7 days of differentiation in the presence of prolactin. Analysis was not performed on cells from the 3-day differentiation assay, nor those treated with steroid hormones due to the lack of easily identifiable neurites after the shorter differentiation period or when cultured in the absence of Phenol Red and 4-OHT. Images were acquired using the CellInsight with a 10× objective as described above. Image analysis was performed using the web-based Columbus Analysis System (Perkin Elmer) using a manually created protocol. Briefly, DAPI-positive nuclei were identified using the predefined “method B”, with the parameters for thresholding, splitting and contrast settings defined to properly identify single nuclei. DAPI-positive nuclei smaller than 30 μm^2^ were excluded from further analysis as they were assumed to be debris. The cytoplasm was identified using the predefined “method C” based upon MAP2 staining. Cells were excluded from further analysis if the cytoplasm was in contact with the field edge or the average MAP2 staining intensity across the cytoplasmic area was below 350 arbitrary units (AU) to only consider MAP2-positive neurons that lay entirely within the field of view. The channel containing MAP2 then underwent a sliding parabola filter step with a curvature value of 20 to remove background signal. Neurites of whole MAP2-positive cells were identified and traced using the CSIRO Neurite Analysis 2 method on the filtered MAP2 channel. Exported parameters were: total neurite length per well, maximum neurite length per well, the average number of neurite roots originating at the cell body per MAP2-postive cell, the average number of neurite extremities per MAP2-postive cell and the average number of branchpoints per MAP2-postive cell.

### Statistical analysis

All statistical analyses were carried out using GraphPad Prism software (version 6.07, GraphPad Software Inc.). For each hormone under investigation, comparisons in cell marker levels between treatment groups were generally carried out using a one-way ANOVA with post hoc comparisons performed using the Bonferroni’s post-hoc test. Statistically significant differences were considered when *p* < 0.05 and are illustrated on graphs, where applicable. The number of biological repeats is indicated for each experiment. To check that the data fit the assumptions for an ANOVA, Brown–Forsyth test and Bartlett’s test were performed. If the data do not fit the assumption of equal variance, the Kruskal–Wallis followed by the Dunn’s multiple comparisons non-parametric tests were used.

## Results

The prolactin receptor is the most highly expressed sex hormone receptor both in hippocampal post-mortem tissue and in the HPC line.

**Fig. 2:**
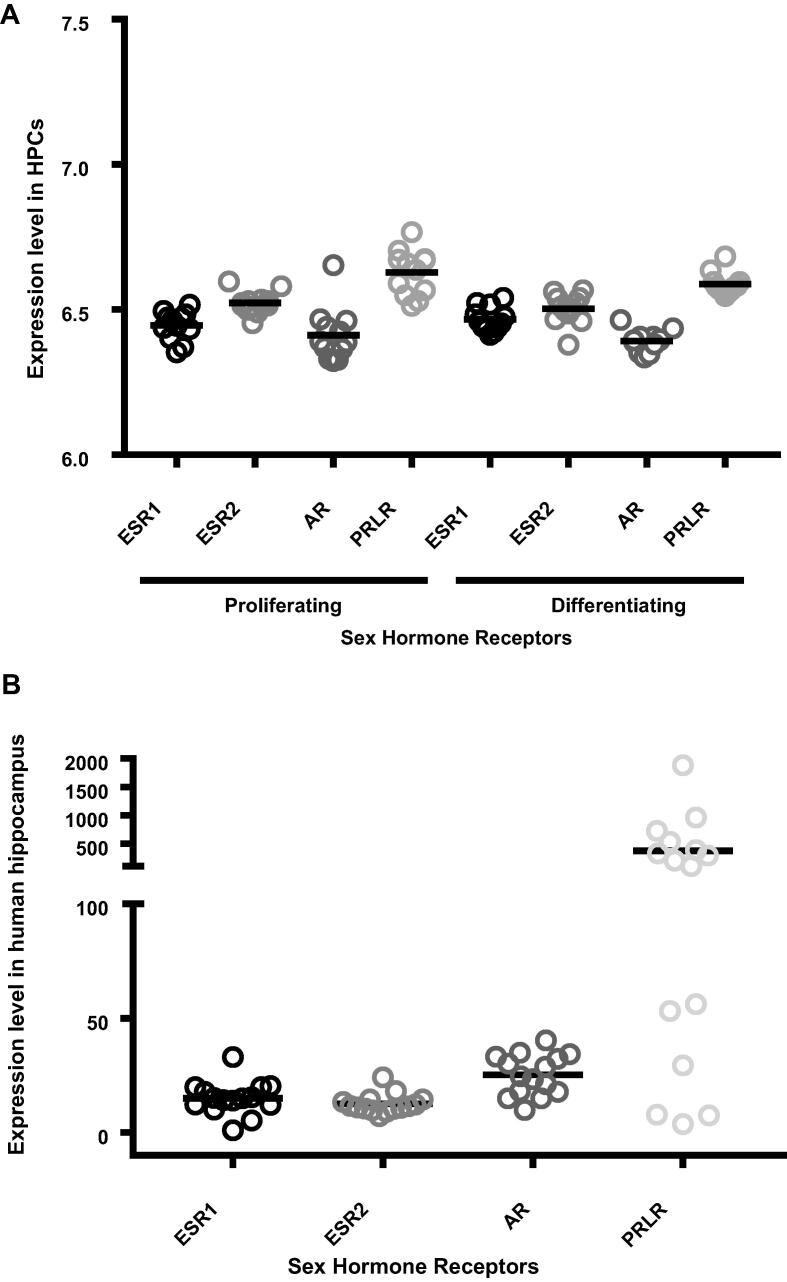
Relative gene expression levels of sex hormone receptors. (**A**) Expression level of each sex hormone in 15 post-mortem hippocampal samples collected from control individuals, and submitted to RNA-sequencing as part of the Stanley Neuropathology Consortium. (**B**) Expression levels of each sex hormone in human hippocampal progenitor cells, submitted to microarray analysis, either whilst they are proliferating (PRO) or after they have differentiated (DIF) for a seven-day period. In both (**A**) and (**B**), the prolactin receptor is expressed more highly than all other receptor types (*p* < 0.005).

Sex hormone receptors were expressed both in post-mortem hippocampal samples and in both proliferating and differentiating HPCs (Fig. 2). ANOVA revealed that there were significant differences in the expression levels of each receptor type in the hippocampus (*F* (3, 56) = 7.273, *p* = 0.0003) and in the progenitor cells (*F* (7, 88) = 25.9, *p* < 0.0001), with PRLR consistently being expressed most highly relative to other transcripts (Bonferroni post hoc test; *p* < 0.005). There were no significant changes in individual receptor level expression when comparing between proliferating and differentiating HPCs (*p* > 0.05).

### Prolactin has no effect on proliferating human HPCs

**Fig. 3:**
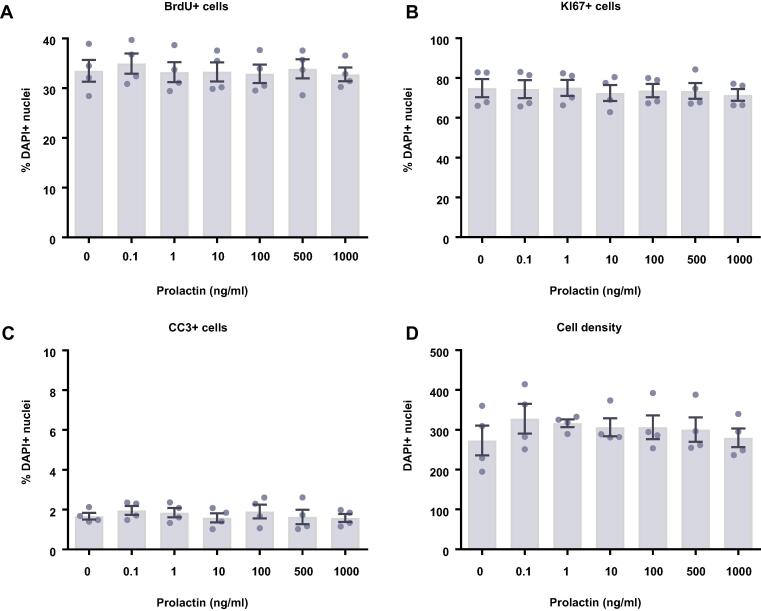

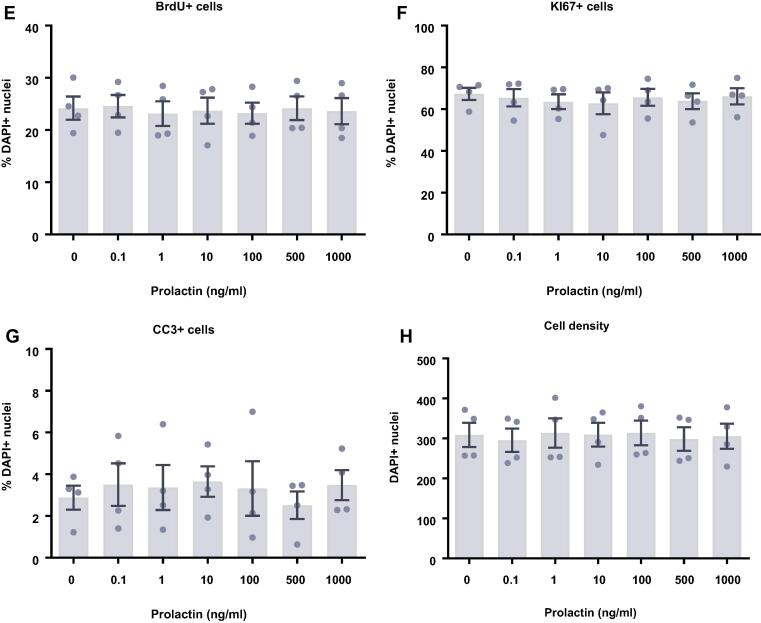
Cellular marker quantification following 24 h or 48 h of proliferation with prolactin. HPCs were treated with differing concentrations of prolactin for 24 h (**A**–**D**) or 48 h (**E**–**H**) while cells were maintained in a proliferative state. Each graph indicates the proportion of DAPI-positive cells staining positively for each marker. (**A**) 24 h prolactin treatment had no impact on the proportion of cells synthesising new DNA as indicated by BrdU staining. (**B**) It also did not change the number of cells in the cell cycle as indicated by KI67 staining. (**C**) There was no difference in the proportion of apoptotic cells as shown by CC3 staining. (**D**) There was also no effect of prolactin treatment on cell density. (**E**) Prolactin treatment had no impact on the proportion of cells synthesising new DNA as indicated by BrdU staining after 48 h. (**F**) It also did not change the number of cells in the cell cycle as indicated by KI67 staining. (**G**) There was no difference in the proportion of apoptotic cells as shown by CC3 staining. (**H**) There was also no effect of prolactin treatment on cell density. Group differences were detected using a one-way ANOVA. Significantly different groups were considered when *p* < 0.05. *N* = 4 for all groups.

We next investigated what effect prolactin treatment had on human HPCs maintained in a proliferative state for either 24 (Fig. 3A–D) or 48 h (Fig. 3E–H). To do so we quantified the proportion of cells staining positively for a range of cellular markers following the treatment period. We found that prolactin treatment had no effect on cell density, markers of proliferation (Ki67), or cells actively undergoing DNA synthesis (BrdU), after either a 24- or 48-h treatment. Similarly, there were no effects on apoptosis (CC3).

### Prolactin decreases proliferation after 7 days of differentiation

**Fig. 4:**
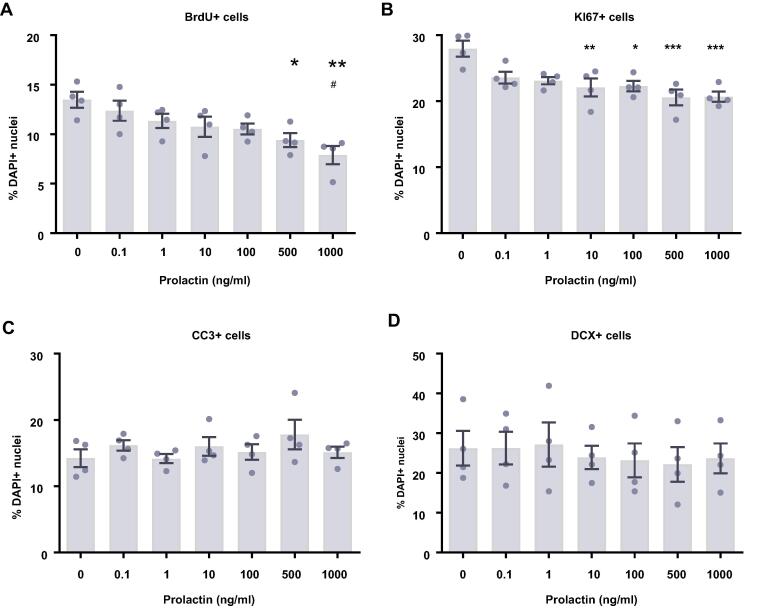

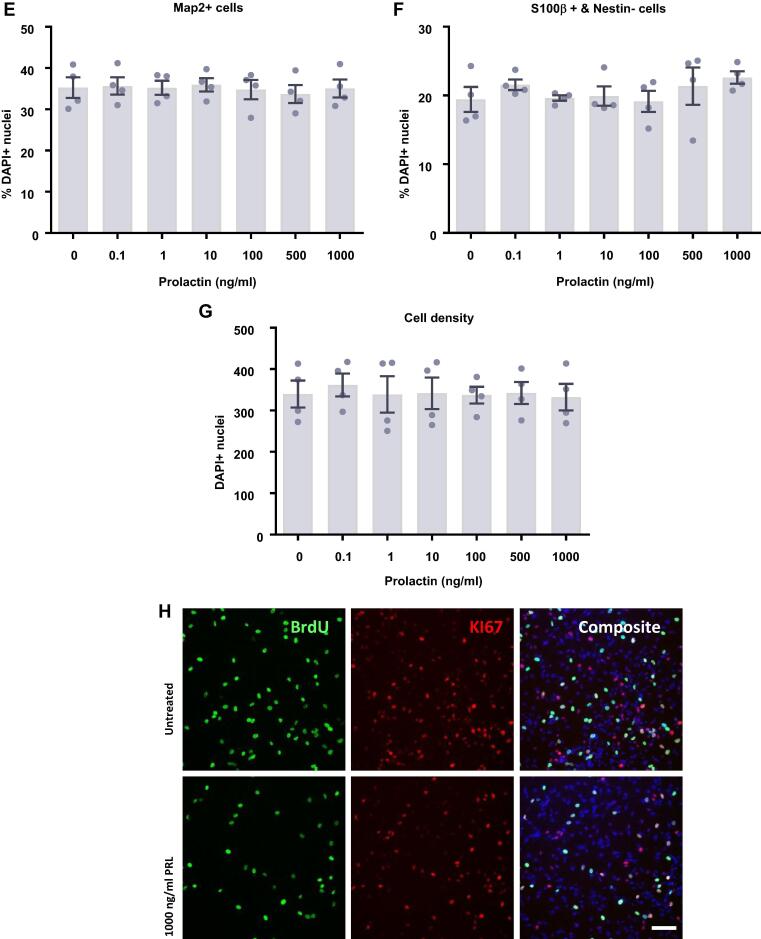
Cellular marker quantification following 7 d of differentiation with prolactin. HPCs were treated with differing concentrations of prolactin during both the 48 h proliferation phase and 7 d differentiation phases of the cellular assay. Each graph indicates the proportion of DAPI-positive cells staining positively for each marker. (**A**) Prolactin treatment decreased the proportion of cells synthesising new DNA as indicated by BrdU staining. (**B**) It also decreased the number of cells in the cell cycle as indicated by KI67 staining. Together this indicates that prolactin decreases HPC proliferation. (**C**) There was no difference in the proportion of apoptotic cells as shown by CC3 staining. (**D**) Prolactin treated had impact on the proportion of cells staining positively for DCX or (**E**) MAP2 indicating no change in neuronal differentiation. (**F**) There was also no effect of prolactin treatment on astrocytic cells which were positive for S100β but negative for nestin. (**G**) There was also no effect of prolactin treatment on cell density. Group differences were detected using a one-way ANOVA followed by Bonferroni’s post-hoc test. Significantly different groups were considered when *p* < 0.05. *Indicates a significant difference compares to 0 ng/ml. ^#^Indicates a significant difference compares to 0.1 ng/ml. *N* = 4 for all groups. **H.** Representative images of HPCs stained for BrdU and Ki67 with and without prolactin treatment. Scale bar = 100 μm.

We next examined the effect of prolactin treatment on HPCs undergoing 7 days of differentiation (Fig. 4). We observed that increasing the concentration of prolactin decreased the proliferative capacity of HPCs, exhibited by KI67-positive cells (*F*(6,21) = 6.19, *p* = 0.0007; Fig. 4B). Bonferroni’s post-hoc test revealed significant decreases in KI67-positive cells in the 10, 100, 500 and 1000 ng/ml treatment groups (*p* < 0.05) compared to control groups. Similarly, prolactin treatment decreased the proportion of cells synthesizing new DNA as indicated by BrdU incorporation in the final 4 hours of the assay (*F*(6,21) = 4.91, *p* = 0.0028; Fig. 4A). Significant differences were detected between the control group and the 500 and 1000 ng/ml treatment groups (*p* < 0.05), as well as between the 0.1 ng/ml and 1000 ng/ml treatments (*p* < 0.05). There were no significant effects of prolactin treatment upon neuronal differentiation (DCX+ or MAP2+ cells) or astrocytic differentiation (S100β+ nestin− cells). Prolactin also had no impact on apoptosis (CC3+ cells) or cell density.

**Fig. 5:**
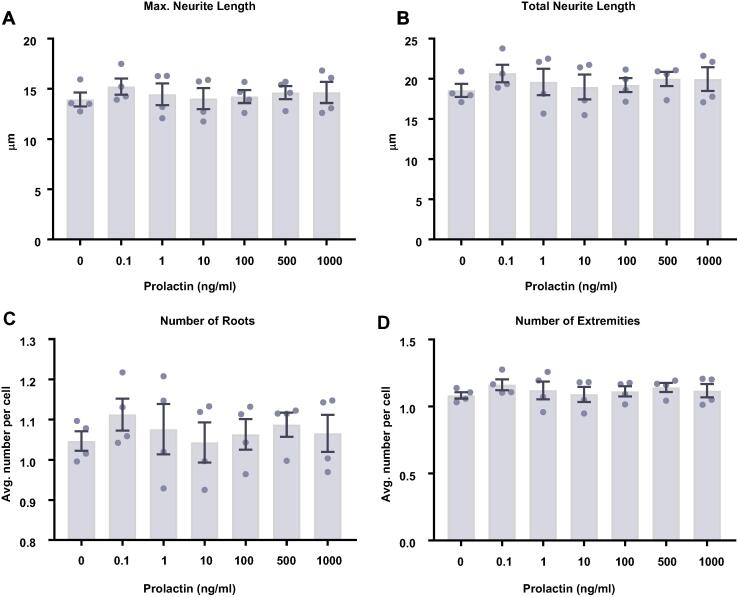

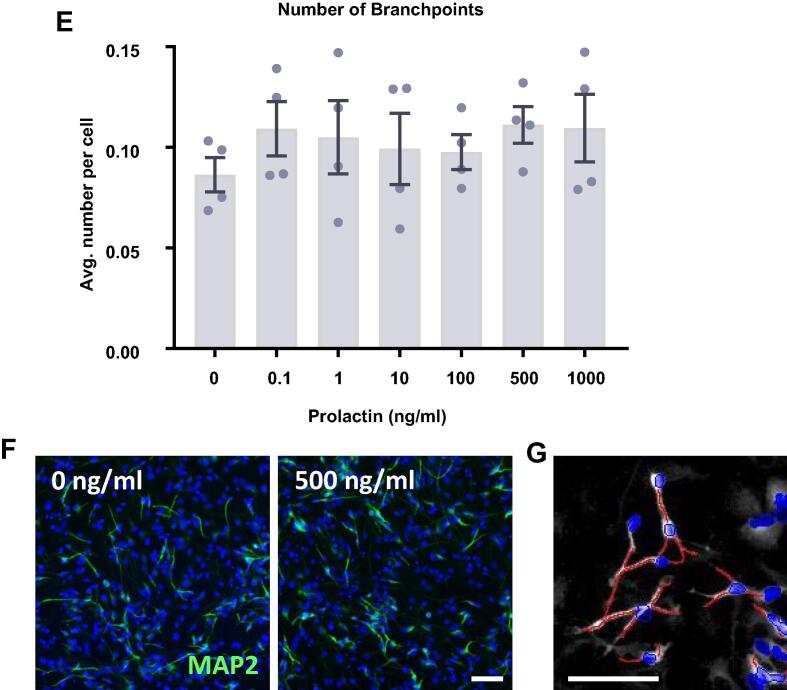
Neurite outgrowth analysis of prolactin-treated hippocampal progenitor cells after 7 days of differentiation. Following 7 d of differentiation in the presence of prolactin, cells were co-stained for MAP2 and DAPI. Columbus software was used to identify neurons based upon nuclear DAPI staining and cytoplasmic MAP2 staining. The neurites of MAP2+ neurons were traced and the number, length and branching were quantified. (**A**) Prolactin had no effect on the maximum neurite length present in each condition. (**B**) Prolactin did not impact the total length of neurites traced in each well. (**C**) There was no impact on the number of roots corresponding to the number of neurites originating at the cell body border, calculated as the average number per cell. (**D**) There was also no impact on the number of extremities corresponding to the number of ends of individual neurites, calculated as the average number per cell. (**E**) Prolactin also had no impact on the number of nodes or points of intersection between two or more neurite segments, calculated as the average number per cell. Group differences were detected using a one-way ANOVA. Significantly different groups were considered when *p* < 0.05. *N* = 4 for all groups. (**F**) Representative images of the 0 and 500 ng/ml prolactin treatment groups illustrating the similar neurite outgrowth. DAPI nuclear stain shown in blue and MAP2 shown in green. (**G**) Example of the automated neurite tracing performed by Columbus. Group differences were detected using a one-way ANOVA. Significantly different groups were considered when *p* < 0.05.

At this stage, the cells were sufficiently differentiated to perform morphological analysis on neuronal cells to identify any changes in neuronal morphological maturation (Fig. 5). We found no effect of prolactin treatment upon the length of neurites as indicated by the maximum neurite length present in each condition or the total neurite length in each condition. There was also no impact on the number of neurites as indicated by the average number of neurite roots originating at the cell body or the average number of neurite extremities. Finally, there was no impact of prolactin treatment upon the branching of neurites as indicated by the average number of branchpoints. Therefore, it appears that prolactin treatment has no impact on the number, length or branching of neurites.

### Prolactin increases neuronal differentiation after 3 days of differentiation

After 7 days of differentiation, prolactin treatment unexpectedly decreased the proliferative capacity of the cells, with no observed change in differentiation. We hypothesized that this was due to an earlier impact on differentiation. We therefore investigated the impact of prolactin upon HPCs which underwent a shorter period of differentiation of 3 days, similar to that used in the only other published investigation of prolactin upon human neural stem cells (Pathipati et al., 2011).

**Fig. 6:**
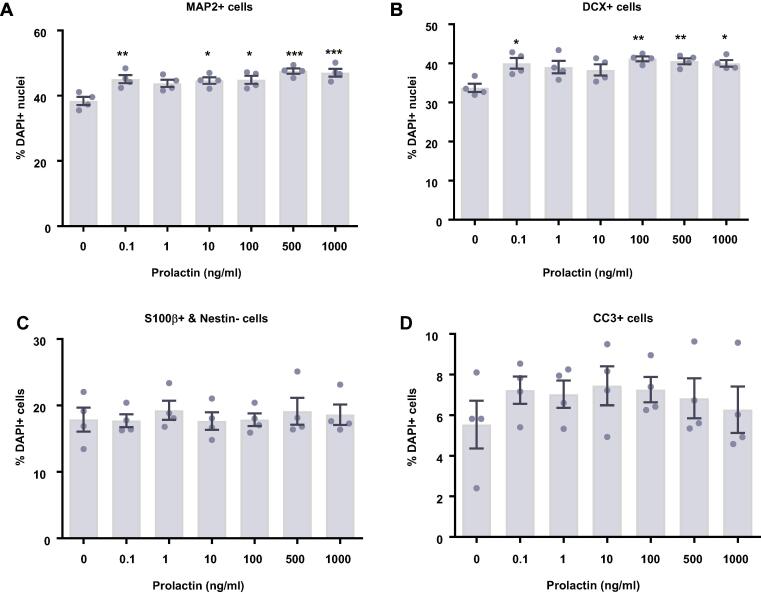

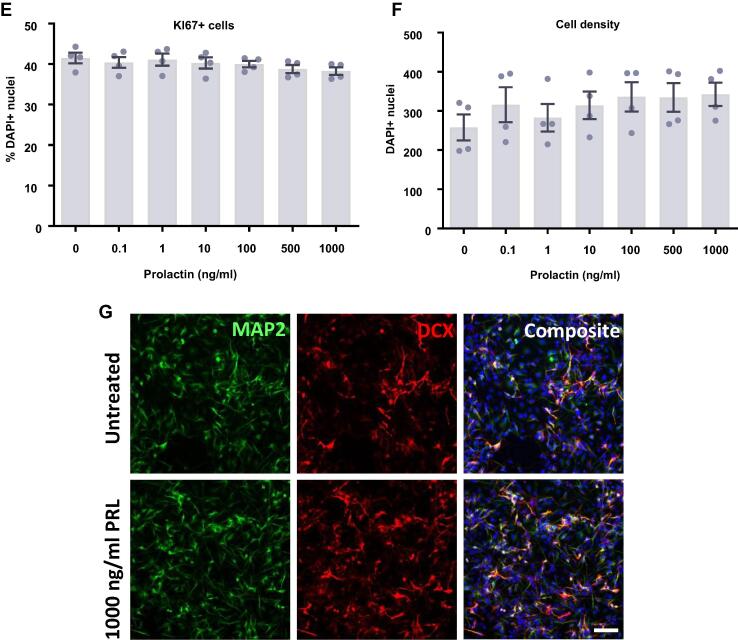
Cellular marker quantification following 3 d of differentiation with prolactin. HPCs were treated with differing concentrations of prolactin during both the 48 h proliferation phase and 3 d differentiation phases of the cellular assay. Each graph indicates the proportion of DAPI-positive cells staining positively for each marker. (**A**) Prolactin treated increased the proportion of cells staining positively for both MAP2 and (**B**) DCX indicating an increase in neuronal differentiation. (**C**) There was no effect of prolactin treatment on astrocytic cells which were positive for S100β but negative for nestin. (**D**) There was no difference in the proportion of apoptotic cells as shown by CC3 staining. (**E**) It also did not impact the number of cells in the cell cycle as indicated by KI67 staining. (**F**) There was also no effect of prolactin treatment on cell density. Group differences were detected using a one-way ANOVA followed by Bonferroni’s post-hoc test. Significantly different groups were considered when *p* < 0.05. *Indicates a significant difference compares to 0 ng/ml. *N* = 4 for all groups. (**G**) Representative images of differentiating cells expressing MAP2 (green) or DCX (red) with or without prolactin treatment. Nuclei stained with DAPI (blue). Scale bar = 100 μm.

Following 3 days of differentiation, prolactin treatment increased the proportion of HPCs gaining a neuronal fate (Fig. 6). There were significant group differences in cells staining positively for DCX (*F*(6,21) = 4.70, *p* = 0.0035; Fig. 6B) and MAP2 (*F*(6,21) = 7.04, *p* = 0.0003; Fig. 6A). Bonferroni post-hoc test revealed a significant increase in DCX-positive cells treated with 0.1, 100, 500 and 1000 ng/ml prolactin (*p* < 0.05) and revealed a significant increase in MAP2-positive cells after 0.1, 10, 100, 500 and 1000 ng/ml prolactin (*p* < 0.05) treatment compared to control-treated groups. There were no significant effects on markers relating to cell density, astrocytes (S100β+ nestin− cells), proliferation (KI67+ cells), or cell death (CC3+ cells). We were unable to perform morphological analysis as this time point due to the immaturity of neuronal cells present in the culture.

### Estradiol and testosterone increase the density of HPCs

**Fig. 7:**
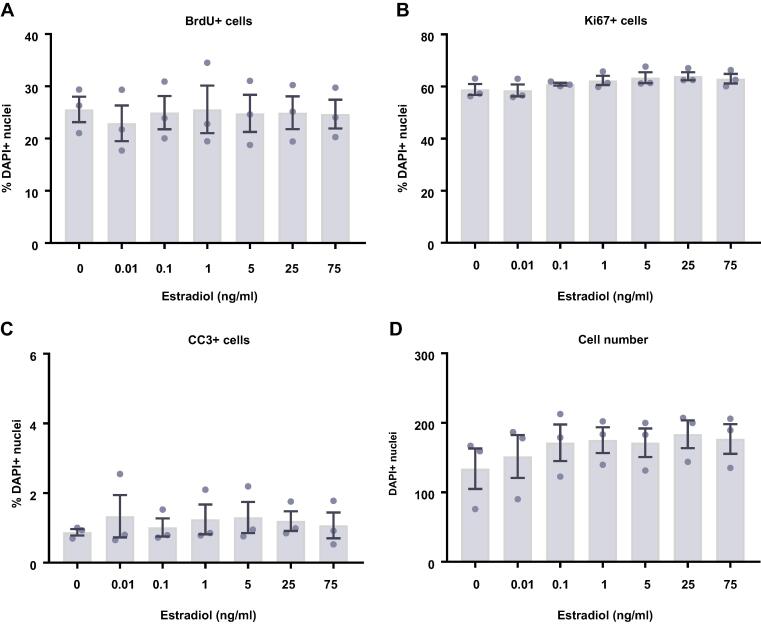

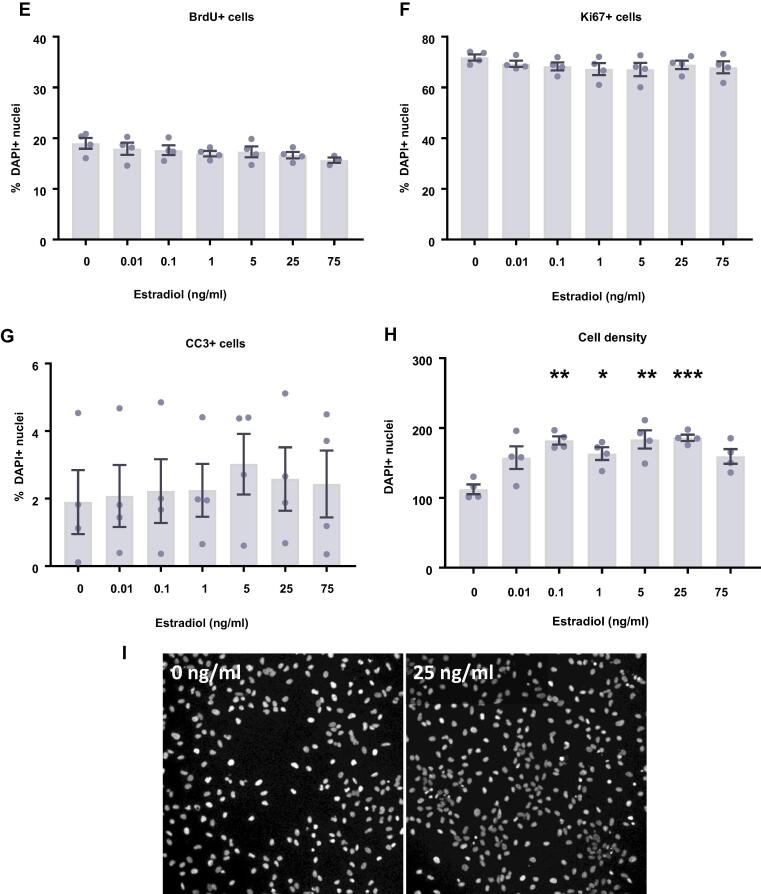
Cellular marker quantification following 24 h and 48 h of proliferation with estradiol. HPCs were treated with differing concentrations of estradiol for 24 h (A–D) or 48 h (E–H) while cells were maintained in a proliferative state. Each graph indicates the proportion of DAPI-positive cells staining positively for each marker. (**A**) 24 h estradiol treatment had no impact on the proportion of cells synthesising new DNA as indicated by BrdU staining. (**B**) It also did not change the number of cells in the cell cycle as indicated by KI67 staining. (**C**) There was no difference in the proportion of apoptotic cells as shown by CC3 staining. (**D**) Despite there being a slight increase in cell density with increasing estradiol concentration, this was not significant. (**E**) Estradiol treatment had no impact on the proportion of cells synthesising new DNA as indicated by BrdU staining after 48 h. (**F**) It also did not change the number of cells in the cell cycle as indicated by KI67 staining. (**G**) There was no difference in the proportion of apoptotic cells as shown by CC3 staining. (**H**) Estradiol increases cell density, particularly treatments of 0.1, 1, 5 and 25 ng/ml. Group differences were detected using a one-way ANOVA followed by Bonferroni’s post-hoc test. Significantly different groups were considered when *p* < 0.05. *Indicates a significant difference compares to 0 ng/ml. *N* = 4 for all groups. (**I**) Representative images of the 0 and 25 ng/ml 48 h estradiol treatment groups illustrating the increase in cell density. Nuclei stained with DAPI. Scale bar = 100 μm.

**Fig. 8:**
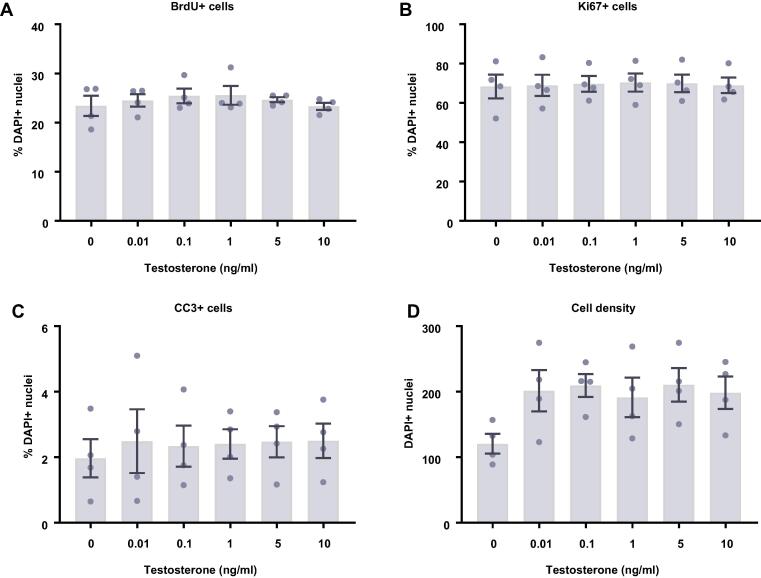

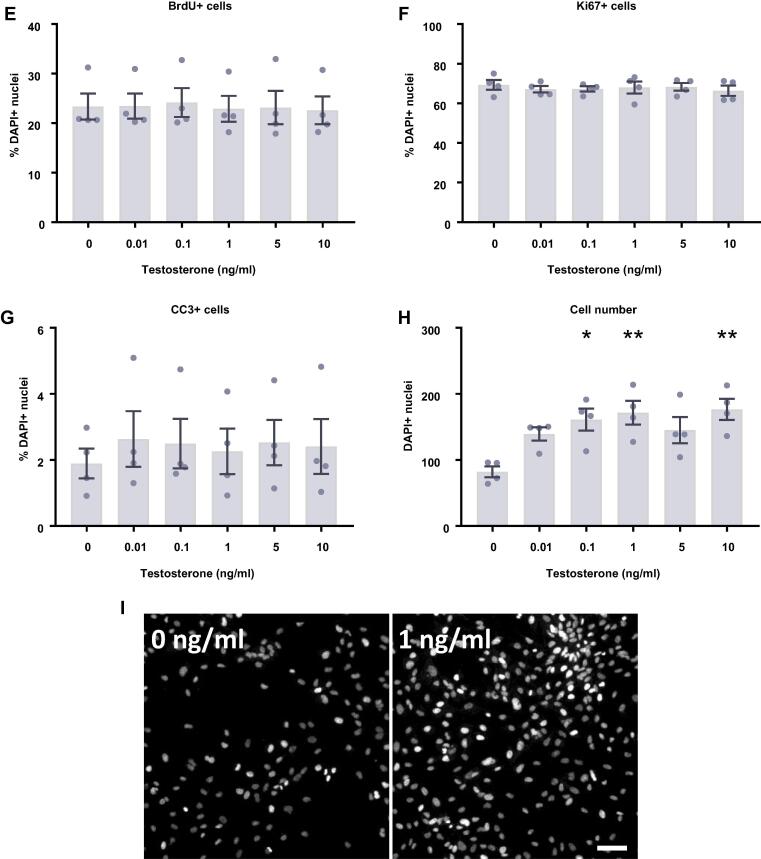
Cellular marker quantification following 24 h and 48 h of proliferation with testosterone. HPCs were treated with differing concentrations of testosterone for 24 h (**A**–**D**) or 48 h (E-H) while cells were maintained in a proliferative state. Each graph indicates the proportion of DAPI-positive cells staining positively for each marker. (**A**) 24 h testosterone treatment had no impact on the proportion of cells synthesising new DNA as indicated by BrdU staining. (**B**) It also did not change the number of cells in the cell cycle as indicated by KI67 staining. (**C**) There was no difference in the proportion of apoptotic cells as shown by CC3 staining. (**D**) Despite there being an increase in cell density with increasing testosterone concentration, this was not significant. (**E**) Testosterone treatment had no impact on the proportion of cells synthesising new DNA as indicated by BrdU staining after 48 h. (**F**) It also did not change the number of cells in the cell cycle as indicated by KI67 staining. (**G**) There was no difference in the proportion of apoptotic cells as shown by CC3 staining. (**H**) Testosterone increases cell density, particularly treatments of 0.1, 1 and 10 ng/ml. Group differences were detected using a one-way ANOVA followed by Bonferroni’s post-hoc test. Significantly different groups were considered when *p* < 0.05. *Indicates a significant difference compares to 0 ng/ml. *N* = 4 for all groups. (**I**) Representative images of the 0 and 1 ng/ml 48 h testosterone treatment groups illustrating the increase in cell density. Nuclei stained with DAPI. Scale bar = 100 μm.

We next investigated the impact of the steroid hormones, estradiol (Fig. 7) and testosterone (Fig. 8) upon proliferating HPCs. After 24 h of treatment there appeared to be a slight increase in cell density with estradiol although these group differences were not statistically significant (*F*(6,14) = 0.51, *p* = 0.79; Fig. 7D). After a further 24 h of estradiol treatment, there was a statistically significant increase in cell density with estradiol treatment (*F*(6,21) = 6.32, *p* = 0.0006; Fig. 7H). Bonferroni’s post-hoc test revealed an increase in cell number when treated with 0.1, 1, 5, and 25 ng/ml estradiol (*p* < 0.05) compared to control treated cultures. There were no significant differences between the other treatment groups. Despite the increase in cell density we observed no changes in markers of proliferation or apoptosis at these time points to explain this observation. There was no difference in HPC staining for KI67 after 24 h or 48 h indicating no difference in the number of cells active in the cell cycle, nor was there any change in cells undergoing DNA synthesis in preparation for cell division as shown by BrdU incorporation after 24 h and 48 h. There was also no treatment effect on caspase-dependent apoptosis as indicated by CC3 staining after 24 h and 48 h.

Overall, we found that testosterone treatment had a very similar impact on HPCs to estradiol treatment (Fig. 8). After 24 h of testosterone treatment there appeared to be a slight increase in cell density although these group differences were not statistically significant (*F*(5,18) = 1.88, *p* = 0.15; Fig. 8D). After a further 24 h of testosterone treatment, there was a statistically significant increase in cell density in response to testosterone treatment (*F*(5,18) = 5.06, *p* = 0.0046; Fig. 8H). Bonferroni’s post-hoc test revealed an increase in cell number with the 0.1, 1, and 10 ng/ml testosterone treatments (*p* < 0.05) compared to control treated cells. As with estradiol treatment, we observed no changes in any other markers to explain this increase in cell density. We observed no hormone-induced changes in the proportion of HPCs staining positively for KI67, BrdU or CC3 after either the 24 h and 48 h timepoint.

### Estradiol and testosterone have no effect on differentiating HPCs

**Fig. 9:**
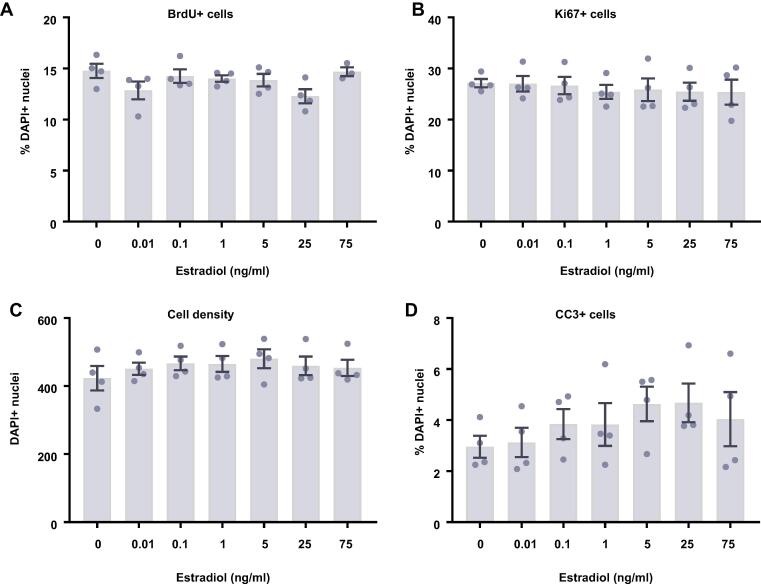

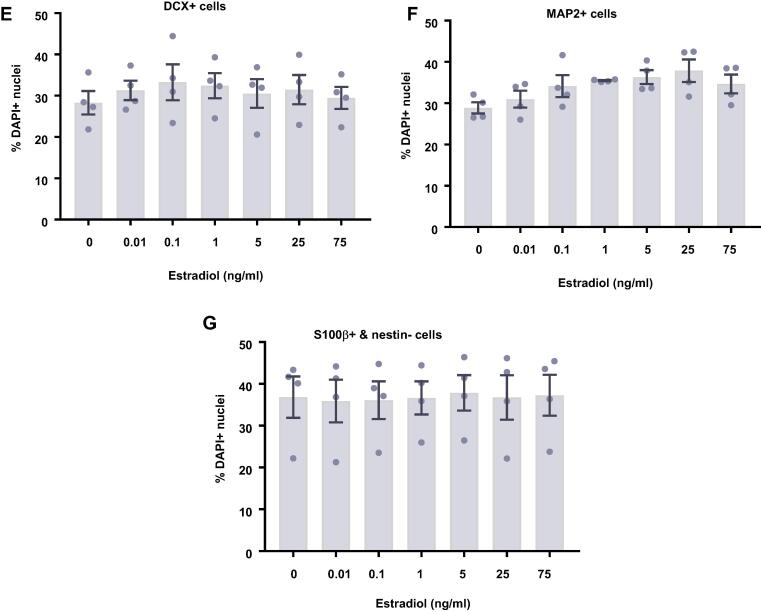
Cellular marker quantification following 7 d of differentiation with estradiol. HPCs were treated with differing concentrations of estradiol during both the 48 h proliferation phase and 7 d differentiation phases of the cellular assay. Each graph indicates the proportion of DAPI-positive cells staining positively for each marker. (**A**) Estradiol treatment had no impact on the proportion of cells synthesising new DNA as indicated by BrdU staining. (**B**) It also did not affect the number of cells in the cell cycle as indicated by KI67 staining. (**C**) There was also no effect of estradiol treatment on cell density. (**D**) There was no difference in the proportion of apoptotic cells as shown by CC3 staining. (**E**) Estradiol treated had no impact on the proportion of cells staining positively for DCX or (**F**) MAP2 indicating no change in neuronal differentiation. (**G**) There was also no effect of prolactin treatment on astrocytic cells which were positive for S100β but negative for nestin. Group differences were detected using a one-way ANOVA followed by Bonferroni’s post-hoc test. Significantly different groups were considered when *p* < 0.05. *N* = 4 for all groups.

**Fig. 10:**
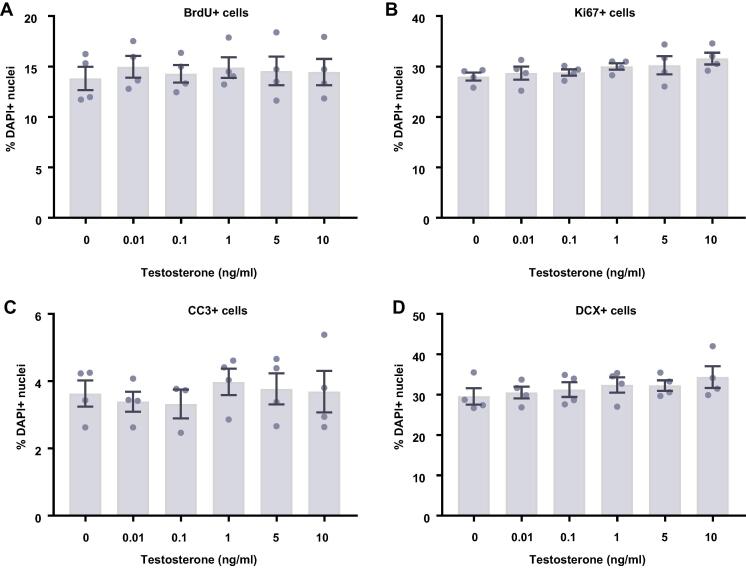

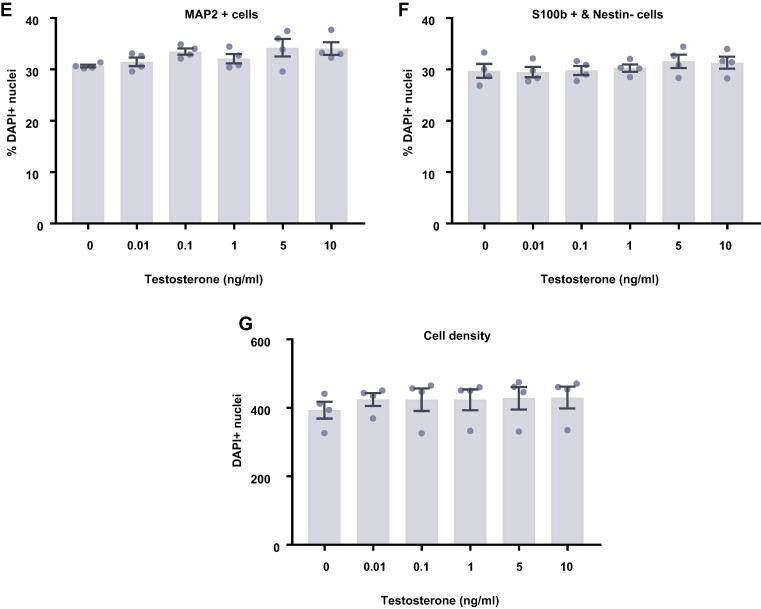
Cellular marker quantification following 7 d of differentiation with estradiol. HPCs were treated with differing concentrations of estradiol during both the 48 h proliferation phase and 7 d differentiation phases of the cellular assay. Each graph indicates the proportion of DAPI-positive cells staining positively for each marker. (**A**) Estradiol treatment had no impact on the proportion of cells synthesising new DNA as indicated by BrdU staining. (**B**) It also did not affect the number of cells in the cell cycle as indicated by KI67 staining. (**C**) There was also no effect of estradiol treatment on cell density. (**D**) There was no difference in the proportion of apoptotic cells as shown by CC3 staining. (**E**) Estradiol treated had no impact on the proportion of cells staining positively for DCX or (**F**) MAP2 indicating no change in neuronal differentiation. (**G**) There was also no effect of prolactin treatment on astrocytic cells which were positive for S100β but negative for nestin. Group differences were detected using a one-way ANOVA followed by Bonferroni’s post-hoc test. Significantly different groups were considered when *p* < 0.05. *N* = 4 for all groups.

In comparison to proliferating HPCs, steroid hormones had no impact on cells undergoing differentiation. Following 7 d of differentiation in the presence of either estradiol (Fig. 9) or testosterone (Fig. 10), there were no group differences observed for the proportion of cells staining positively for DCX or MAP2, indicating no change in neuronal differentiation in response to either hormone. Due to the slower rate of differentiation in the absence of Phenol Red or 4-OHT necessary to perform assays with steroid hormones, we were unable to examine the morphology of these neuronal cells. There was no impact on the proportion of HPCs becoming astrocytes as indicated by S100β-positive and nestin-negative cells. Similarly, there was no change to number of proliferating cells active in the cell cycle as indicated by KI67 staining or change in cells undergoing DNA synthesis in preparation for cell division as shown by BrdU incorporation. There was also no difference in apoptosis as shown by CC3 staining and there was no difference in average cell count between treatment groups.

## Discussion

Here we aimed to investigate the impact that biologically relevant concentrations of sex hormones have on human hippocampal neurogenesis by using human HPCs as an *in vitro* model. We found that the major receptors for each hormone were expressed in both human postmortem hippocampal tissue as well as the HPC model used here, at least at the RNA level. We found that while prolactin does not impact human HPCs in their proliferative state, it does stimulate neuronal differentiation. We found that prolactin increased the neuronal differentiation of HPCs 3 d after the initiation of differentiation, but this was not observed after 7 d. Instead we saw a decrease in proliferating HPCs at this later timepoint. Conversely, while both estradiol and testosterone increased cell density in proliferating HPCs, we did not observe any changes in the associated markers for proliferation or apoptosis to explain this. We also found that the steroid hormones had no impact on HPCs undergoing differentiation.

We observed detectable RNA expression of the major receptor types for testosterone, estrogen and prolactin in human postmortem hippocampal tissue. While expression of ESR1, ESR2 and AR were relatively similar, the expression of PRLR was on average 10-fold higher. Conversely, the expression of these same receptors was relatively similar in both proliferating and differentiating HPCs. Due to the differences in methodology used, we are unable to directly compare the gene expression of the receptors between the tissue types. Additionally, the hippocampal tissue used for RNA-seq includes many more cell types than the resident HPCs which further hinders the comparison. Despite this, the work indicates that both the human hippocampus and the human HPCs used here express the sex hormone receptors required to respond to prolactin, estrogen and testosterone, at least at the RNA level. From this data we are unable to say how this translates into relative protein expression, but we can expect there to be some receptor present, supported by previous reports in the human and rodent dentate gyrus (Tabori et al., 2005, González et al., 2007, Mak and Weiss, 2010).

HPC gene expression was examined in untreated environments, which may not match the temporal expression during the cellular assays used here. Hormone exposure may alter the expression of their receptor throughout the assay span, which could explain the time-dependent impact of prolactin on HPC differentiation. Previous work has shown that sex hormones can regulate the expression of their receptor (Ihionkhan et al., 2002, Gutzman et al., 2004). Additionally, this work assessed the global gene expression of a mixed population of cells, particularly after 7 d of differentiation. This will not account for any intercellular variability in gene expression, which may impact the downstream effects of the hormone. Future work could take a single-cell approach rather than studying entire populations. In addition to the receptors targeted here, there are numerous other receptors and modulatory proteins which can impact sex hormone signaling, particularly the numerous receptors which bind estrogens (Ma et al., 2010, Varlakhanova et al., 2010, Filardo and Thomas, 2012, Tetel and Acharya, 2013). Furthermore, these may also change in expression throughout the assay.

We saw no impact of prolactin treatment on HPCs maintained in a proliferative state at either time point evaluated, similar to that seen with murine hippocampal NSCs (Wagner et al., 2009). A more recent study suggested that prolactin should promote cell proliferation in neural stem cells isolated from the hippocampus (Walker et al., 2012). We may not see the same results for many reasons, including differences in species studied, the culturing method or the assay time scale used. Previous studies which observed a proliferative effect of prolactin, did so when growing the cells as neurospheres (Walker et al., 2012). Here the HPCs are cultured as a monolayer and may respond very differently to exogenous treatments. Although we see no changes after 24 h or 48 h of prolactin treatment, this does not rule out any effects of prolactin after a longer incubation or at a later time point.

After 3 d of differentiation in the presence of prolactin we detected an increase in the proportion of HPCs undergoing neuronal differentiation as detected by staining for DCX and MAP2, similar to the effect prolactin has in other NSC studies (Shingo et al., 2003, Pathipati et al., 2011) and the results observed in some animal models (Torner et al., 2009, Mak and Weiss, 2010). In comparison to the effect of prolactin upon human cortical stem cells (Pathipati et al., 2011), the effect seen here did not differ considerably between the concentrations of prolactin tested. This suggests that changes in circulating prolactin concentration may not have a particularly impactful role in AHN control, at least in the timescale studied here.

In comparison to the results seen after 3 d, following 7 d of differentiation we did not observe any effect of prolactin treatment upon neuronal differentiation as indicated by cellular markers as well as morphological analysis. This indicates that there was no sustained increase in neuronal differentiation after 7 d of differentiation in the presence of prolactin. One could hypothesize that the initial surge in neuronal differentiation after prolactin treatment would result in neurons with a more mature phenotype. This was not supported by our neurite outgrowth analysis which found no change in the number, length or branching of neurites, indicating no differences in morphological maturity.

Instead we observed a decrease in proliferation as indicated by a reduction in BrdU incorporation and KI67 immunoreactivity. This decrease in proliferative capacity appeared to be proportional to the prolactin treatment concentration used, although not statistically significant. This indicates that differing concentrations of prolactin, such as those produced by stress or pregnancy may have different effects in the longer term (Lennartsson and Jonsdottir, 2011, Hu et al., 2017). It is unclear why prolactin treatment has resulted in this reduction in proliferative cells, especially as there are no reports of prolactin hindering cell proliferation in the literature, although we hypothesize that this may be due to a greater proportion of post-mitotic neurons in the prolactin-treatment conditions being “pushed” earlier towards neuronal differentiation.

In the work conducted here we observe a significant increase in HPC density when cultured with either estradiol or testosterone, which is clear after 48 h of treatment, but becomes apparent as early as 24 h post-treatment. Overall, the observed increase in cell density supports the work carried out in numerous animal and cellular studies, which also show an increase in dentate gyrus cell number, volume or HPC number (Barha et al., 2009, Duarte-Guterman et al., 2019, Hamson et al., 2013, Ransome and Boon, 2015b, Spritzer and Galea, 2007, Wang et al., 2008, Zhang et al., 2016). Despite the increase in cell density, we found no change in markers of proliferation or caspase-dependent apoptosis to explain this observation. This is unexpected due to the widely reported increases in either KI67-positive cells or BrdU incorporation in response to steroid hormones in *in vivo* and *in vitro* models of AHN (Tanapat et al., 1999, Ormerod et al., 2003, Zhang et al., 2016). These results suggest that the increase in cell number is occurring either through a mechanism missed by the particular markers used in these experiments, such as non-caspase-dependent apoptosis, or an action at a time point not studied here. To account for the increase in cell number, there must either be an increase in proliferation or survival, both of which have been observed in other studies, which remains to be identified. In addition, it would be pertinent to identify whether this increase in cell density is occurring through similar or differing mechanisms between the two hormones. While estradiol often increases cell proliferation (Tanapat et al., 2005, Green and Galea, 2008), testosterone more commonly increases cell survival (Spritzer and Galea, 2007, Hamson et al., 2013), resulting in an increased HPC pool. Here we found that all the estradiol and testosterone treatments increased cell density to some extent, although there is a small amount of variation between the concentrations used. This is reminiscent of previous research, which indicate that the impact of estradiol in the rat hippocampus is dose-dependent, with low and high concentrations increasing proliferation, while mid-range and very high concentrations do not (Tanapat et al., 1999, Barha et al., 2009). The cell density results presented here somewhat match this pattern, although the exact concentrations used are not identical. This may indicate a dose-dependent impact of estradiol on human HPCs, although we did not detect significant differences.

After 7 d of differentiation, there was no change to any cellular phenotype studied in the presence of estradiol or testosterone compared to untreated cells, which is similar to previous reports (Tanapat et al., 2005, Barker and Galea, 2008, Green and Galea, 2008), although other studies indicate that these hormones potentiate the neuronal differentiation of hippocampal NSCs (Saravia et al., 2007, McClure et al., 2013, Zhang et al., 2016) (Duarte-Guterman et al., 2019, Quartier et al., 2018, Ransome and Boon, 2015b). It is unclear why we do not observe an impact on differentiation, particularly into neuronal lineages. It may be that testosterone and estradiol impact differentiation at a later stage which is not picked up due to the relative immaturity of HPC-derived neurons after 7 d of differentiation. Additionally, those studies that do indicate an increase in neuronal differentiation, often explain this through increased survival or proliferation of HPCs in general, leading to a greater number of newborn neurons, rather than a potentiation of differentiation itself (Green and Galea, 2008, Chan et al., 2014). Future work could attempt to investigate these various mechanisms through a combination of early BrdU incorporation and ICC of neuronal and astrocytic markers. This could reveal some interesting results which are missed by only labelling the cultures at the assay endpoint. Furthermore, some studies indicate that multiple weeks are required to be able to observe an increase in estradiol-induced neuronal differentiation (Li et al., 2011), therefore the assay length we have used would not be sufficiently long enough to observe these effects. While we observed no significant impact of estradiol on HPC differentiation, we only quantified the proportion of neurons or astrocytes, ignoring any differentiation into oligodendrocytes due to the very low conversion rate (Anacker et al., 2011). Previous work has demonstrated that estradiol can promote the differentiation of rat NSCs into oligodendrocyte lineages without any concurrent effect on differentiation into a neurons (Okada et al., 2008, Okada et al., 2010). It possible that estradiol could impact oligodendrocyte differentiation without noticeably impacting any other markers.

At this differentiation time point studied, we also no longer observed the increase in cell density that estradiol and testosterone induced in the shorter proliferation assays. It would be tempting to compare this lack of difference in cell density to the suppression of cell proliferation observed in animals in the long-term following estradiol treatment (Ormerod and Galea, 2003, Ormerod et al., 2003). While it is possible a similar mechanism may be involved in both observations, the more likely explanation is that the *in vitro* methodology used here leads to saturation of cell confluence after 10 days in culture and which leads to a suppression of proliferation.

Despite interesting results, this study was subject to a number of limitations. Although the *in vitro* model used here allowed us to study the impact of sex hormones upon HPCs in a wholly human system, it is subject to its own intrinsic limitations. It only considers the direct impacts of sex hormones upon HPCs in these defined conditions and is missing some of the key aspects of the neurogenic niche such as microglia, mature neurons and the cells of the vasculature (Nicola et al., 2015). Secondly, the design of the assays here are relatively short and utilizes only one or two treatments of each hormone. In pregnancy, sex hormones levels remain elevated for much more sustained periods of time (Schock et al., 2016, Hu et al., 2017). Additionally, prolactin is subject to short pulses of increased release in response to, for example, breastfeeding or stressors (Lennartsson and Jonsdottir, 2011, Stuebe et al., 2015), while circulating prolactin, estradiol and testosterone levels fluctuate with the menstrual cycle (Allshouse et al., 2018). Therefore, the method of administration used here may not fully recapitulate the specific chronic nor the dynamic effects that hormones may exert upon AHN. Indeed, chronic estradiol supplementation in mice only promotes HPC proliferation when given daily (Tanapat et al., 2005, Barker and Galea, 2008, McClure et al., 2013, Chan et al., 2014), while recapitulating glucocorticoid hormone oscillations *in vitro* differentially impacts HPC proliferation when compared to continuous treatment (Schouten et al., 2019). Finally, while progesterone is a constant factor in all culture conditions, it may interfere with any impact of steroid hormones. Rodent studies indicate that progesterone may interfere with the proliferative effect that estradiol has in the dentate gyrus (Tanapat et al., 2005). It is therefore possible that in these experiments, some potentiating effects of estradiol or testosterone may be masked by the presence of progesterone. A further problem with the inclusion of progesterone is that it provides the means for the HPCs for synthesize and secrete their own estradiol and testosterone (Hanukoglu, 1992).

The work presented here aimed to characterize the impact of different concentrations of prolactin, estradiol and testosterone upon human HPCs used to model hippocampal neurogenesis. While we see no impact of prolactin upon proliferative HPCs, there is a time-dependent effect on neuronal differentiation. Importantly, we rarely detected differences between different concentrations of prolactin indicating that changes in relative prolactin concentration may not have a particularly impactful effect on the neurogenic process. We found that both estradiol and testosterone have large effects on the cell density of treated HPCs. While there is some indication of a dose-dependent effect of each hormone on cell density, this requires further confirmatory work. Despite these substantial increases in cell number we observe no treatment-related changes in proliferation or apoptosis to account for these phenotypes. We also found no convincing evidence for an impact of steroid hormones on the differentiation of HPCs. Overall, this work provides evidence that sex hormones can impact human hippocampal neurogenesis, at least in the short term. Whether this leads to long-lasting changes to the neurogenic process or not, still needs to be confirmed. Additionally, further work is needed to fully understand the impact of sex hormones upon hippocampal neurogenesis and whether it has a biologically meaningful effect, especially in times of altered circulating sex hormones such as the perinatal period, stress and depressive disorders.
